# Exploration of the Core Pathways and Potential Targets of Luteolin Treatment on Late-Onset Depression Based on Cerebrospinal Fluid Proteomics

**DOI:** 10.3390/ijms24043485

**Published:** 2023-02-09

**Authors:** Kaige Liu, Huizhen Li, Ningxi Zeng, Bozhi Li, Gaolei Yao, Xiaofeng Wu, Hanfang Xu, Can Yan, Lili Wu

**Affiliations:** 1 Research Center of Basic Integrative Medicine, School of Basic Medical Science, Guangzhou University of Chinese Medicine, Guangzhou 510006, China; 2 Key Laboratory of Depression Animal Model Based on TCM Syndrome, Key Laboratory of TCM for Prevention and Treatment of Brain Diseases with Cognitive Dysfunction, Jiangxi University of Chinese Medicine, Nanchang 330004, China

**Keywords:** late-onset depression, luteolin, cerebrospinal fluid, proteomics, axon guidance

## Abstract

Cognitive deficiency is one of the fundamental characteristics of late-onset depression (LOD). Luteolin (LUT) possesses antidepressant, anti-aging, and neuroprotective properties, which can dramatically enhance cognition. The altered composition of cerebrospinal fluid (CSF), which is involved in neuronal plasticity and neurogenesis, directly reflects the physio-pathological status of the central nervous system. It is not well known whether the effect of LUT on LOD is in association with a changed CSF composition. Therefore, this study first established a rat model of LOD and then tested the therapeutic effects of LUT using several behavioral approaches. A gene set enrichment analysis (GSEA) was used to evaluate the CSF proteomics data for KEGG pathway enrichment and Gene Ontology annotation. We combined network pharmacology and differentially expressed proteins to screen for key GSEA–KEGG pathways as well as potential targets for LUT therapy for LOD. Molecular docking was adopted to verify the affinity and binding activity of LUT to these potential targets. The outcomes demonstrated that LUT improved the cognitive and depression-like behaviors in LOD rats. LUT may exert therapeutic effects on LOD through the axon guidance pathway. Five axon guidance molecules—*EFNA5*, *EPHB4*, *EPHA4*, *SEMA7A*, and *NTNG*—as well as *UNC5B*, *L1CAM*, and *DCC*, may be candidates for the LUT treatment of LOD.

## 1. Introduction

Population aging is becoming a more serious societal concern worldwide. Late-onset depression (LOD), a specific subtype of late-life depression, is defined as a primary depression that first manifests after the age of 60 [1]. Numerous studies have demonstrated that patients with LOD have a higher risk of cognitive deficits [2] and dementia [3], exhibiting a reduced processing speed and executive ability [4]. Patients with LOD suffer from poor quality of life and high rates of disability and mortality, aggravating the health care burden on communities [5]. Consequently, identifying the pathogenic pathways and potential targets of LOD is of the utmost importance.

Cerebrospinal fluid (CSF) is the biological fluid that is closest to the brain, and changes in its composition can directly reflect pathophysiological changes in the central nervous system (CNS) [6]. Essential neurological processes such as neuronal plasticity, immunological regulation, and neurogenesis are modulated by peptides and proteins found in the CSF [7]. It is demonstrated that the proteomics of the CSF is altered in depressed individuals and that proteins with differential expression are directly linked to CNS dysfunction and damage [8]. The aging brain sends signals to the CSF that interfere with the production of neurotrophic molecules, leading to a decline in cognition and neurogenesis in the hippocampus [9]. 

Luteolin (LUT) is a native flavonoid that is widely distributed in nature. For instance, LUT is found in olive oil, oranges, and carrots, as well as in herbs such as scutellaria baicalensis, purple perilla, and honeysuckle. Notably, the peripheral administration of LUT is capable of freely penetrating the blood–brain barrier to provide neuroprotective effects against brain damage [10]. In other words, LUT is able to reach the disease site in the presence of neurological abnormalities and acts directly on the place where the lesion occurs [11]. In addition, the consumption of foods high in LUT content has been linked to a lower chance of developing chronic diseases, according to epidemiological studies [12]. The regular intake of dietary supplements containing LUT helps to suppress or prevent stress-induced adverse effects [13]. Previous research has shown that LUT possesses a variety of pharmacological activities, such as anti-inflammation, anti-oxidation, anti-aging, neuroprotective, neurotrophic, and neurogenic actions [14,15]. LUT can enhance or even rehabilitate hippocampal neurogenesis, as well as promote dendritic spine maturation and reduce hippocampal neuronal death, thus providing neuroprotective effects against brain injury [16]. 

Proteins are the functional components that drive most cellular processes. Many drugs exert their pharmacological properties by interacting with target proteins. Identifying the drug-specific protein targets is a vital step in revealing their action mechanisms, thereby strengthening our understanding of drug pharmacodynamics [17]. The emergence and continuous evolution of proteomics technologies have fostered our knowledge of CSF constituents. In addition, proteomics allows for the identification and comprehensive characterization of cellular targets, providing insight into the action mechanisms of drugs [18]. 

In this project, we first established an LOD rat model and detected the therapeutic effect of LUT on LOD rats through a series of behavioral measurements. Subsequently, CSF proteomics data were analyzed using the gene set enrichment analysis (GSEA) method for KEGG pathway enrichment. We also combined network pharmacology and differentially expressed proteins to screen the key GSEA–KEGG pathway as well as potential targets of LUT for LOD treatment. Molecular docking was performed to verify the affinity and binding activity of LUT to these potential targets. In summary, the present work provides a foundation for the treatment scheme of LOD and the clinical application of LUT.

## 2. Results

### 2.1. LUT Could Improve Depression-like Behaviors in LOD Rats

**Figure 1:**
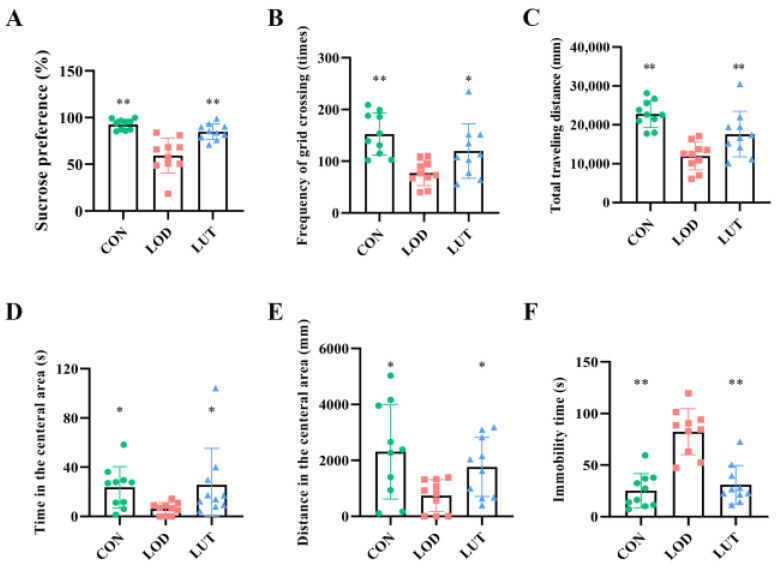
Luteolin (LUT) could improve depression-like behaviors in late-onset depression (LOD) rats. (**A**) Results of sucrose preference (%) (n = 10); (**B**–**E**) results for the open field test (n = 10); (**B**) frequency of grid crossings (times); (**C**) total distance; (**D**) time spent in the central area (s); (**E**) distance traveled in the central area (mm); and (**F**) the mobility time in the forced swimming test (n = 10). All data are expressed as mean ± SEM. ** *p* < 0.01, * *p* < 0.05, compared with LOD group.

Compared with the CON group, the sucrose preference, the number of grid crossings and the total distance traveled were all reduced in LOD rats (*p* < 0.01). Likewise, LOD rats showed a decrease in the distance and time traveled in the central area in the open field test (OFT) (*p* < 0.05) and a significant increase of immobility time in the forced swimming test (FST) (*p* < 0.01). When treated with LUT, rats showed an increase in sucrose preference, number of grid crossings, and total distance traveled, as well as a decrease in immobility time. In comparison with the LOD rats, the distance and time spent in the central zone of the OFT were increased in the LUT rats. This indicated that LUT significantly ameliorated depression, anxiety, and despair in LOD rats (Figure 1).

### 2.2. LUT Could Improve the Cognition of LOD Rats

**Figure 2:**
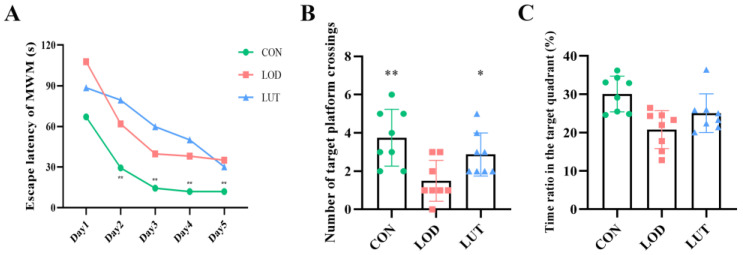
LUT could improve cognition of LOD rats. (**A**) The escape latency in the orientation navigation test (s) (n = 8). (**B**) The number of target platform crossings in spatial exploration test (times) (n = 8). (**C**) Time ratio in the target quadrant in spatial exploration test (%) (n = 8). All data are expressed as mean ± SEM. ** *p* < 0.01, * *p* < 0.05, compared with LOD group.

The results of the orientation navigation experiment in the Morris water maze test (MWM) showed that the group, time, and group * time satisfied Mauchly’s sphericity test (*p* > 0.05). Tests for within-subject effects disclosed that the interaction of group and time had no significant effect on the escape latency of rats (*p* > 0.05). Statistical significance was observed in the main effects for group (*p* < 0.01). The escape latency was shorter in the CON group than in the LOD group (*p* = 0.004). Similarly, the main effect for time reached statistical significance (*p* < 0.01). On days 2–5, the escape latency was significantly lower in the CON group than in the LOD group (*p* < 0.01) (Figure 2A).

Spatial exploration experiments illustrated that LUT exerted a beneficial effect on improving memory function in LOD rats. The number of platform crossings in LOD rats was markedly inferior to the number of platform crossings in CON rats (*p* < 0.01). Following LUT treatment, the number of platform crossings and the time ratio of the quadrant in which the platform was located rose in LOD rats (Figure 2B,C).

### 2.3. Analysis of GSEA–KEGG Pathway in LOD Treatment with LUT

#### 2.3.1. A General Description of CSF Proteomics Data

**Figure 3:**
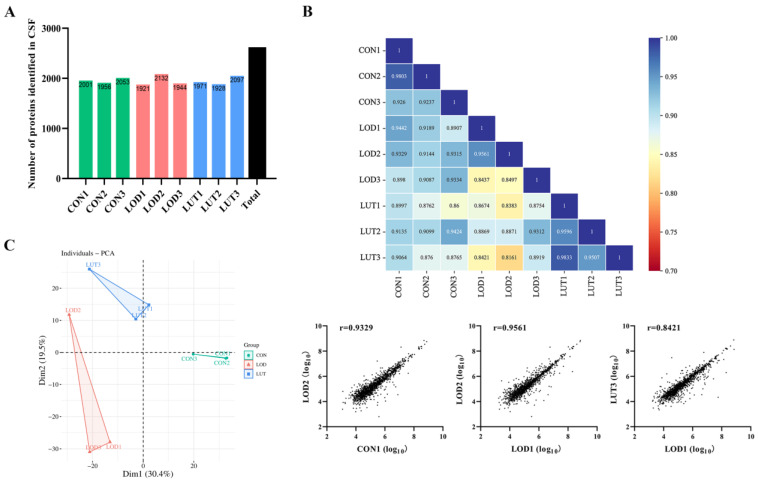
A general description of cerebrospinal fluid (CSF) proteomics data. (**A**) The number of proteins identified in each group of CSF (n = 3). (**B**) Correlation heat map and scatter plots of CSF protein data for each sample (n = 3). (**C**) Principal component analysis plot displaying the intergroup variability of CSF proteomics data for each sample.

The proteins of each sample in the CON, LOD, and LUT groups were examined using data independent acquisition (DIA) proteomics technology, and a total of 2620 proteins were recognized (Figure 3A). 

A correlation analysis of the samples from the CON, LOD and LUT groups revealed high correlation coefficients both within and between groups, indicating that the samples were reproducible (Figure 3B).

A principal component analysis (PCA) was employed to observe the distribution of CSF proteomics data in the CON, LOD, and LUT groups. The results presented that the within-group variability of the samples in the three groups was minor, while the between-group differences were distinct. Of these differences, the differences between samples in the CON and LOD groups were the largest, whereas the samples in the CON and LUT groups appeared relatively similar (Figure 3C).

**Figure 4:**
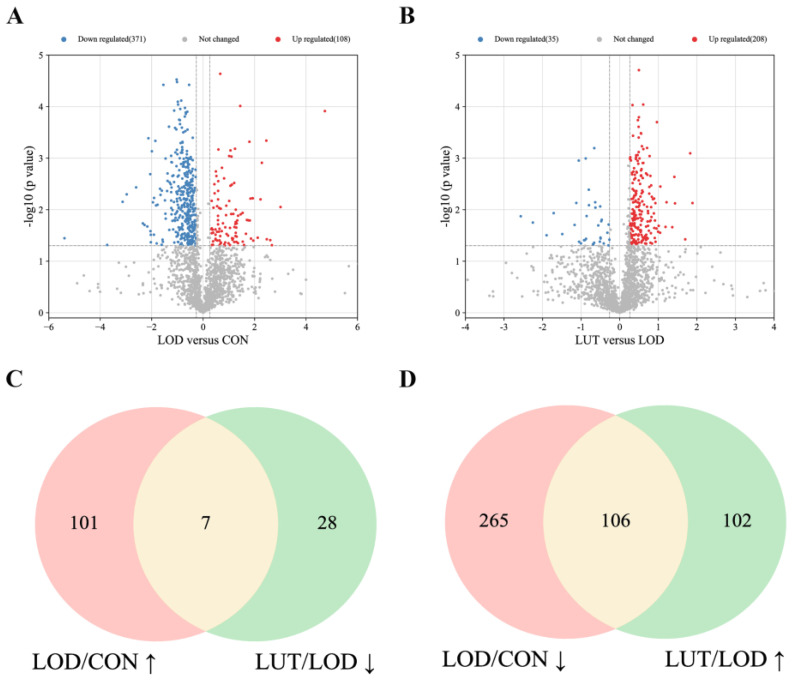
Differentially expressed proteins (DEPs) in CSF proteomics data. (**A**) DEPs between LOD and CON groups, with red indicating upregulation and blue indicating downregulation. (**B**) DEPs between LUT and LOD groups, with red indicating upregulation and blue indicating downregulation. (**C**) Venn diagram showings that seven proteins were upregulated in LOD group (vs. CON) and simultaneously downregulated in LUT group in total (vs. LOD). (**D**) Venn diagram displaying that 106 proteins were downregulated in LOD group (vs. CON) and upregulated in LUT group (vs. LOD).

When compared to the CON rats, 108 proteins were significantly upregulated and 371 proteins were dramatically downregulated in the CSF of LOD rats (Figure 4A). In contrast to the LOD rats, 208 proteins were remarkably upregulated and 35 proteins were significantly downregulated in the CSF of LUT rats (Figure 4B).

Among all the DEPs, seven proteins were upregulated in the LOD group (vs. the CON group) and simultaneously downregulated in the LUT group in total (vs. the LOD group) (Figure 4C). Similarly, a series of 106 proteins were downregulated in the LOD group (vs. the CON group) and upregulated in the LUT group (vs. the LOD group) (Figure 4D). Collectively, these 113 proteins were regarded as differentially expressed proteins for the treatment of LOD by LUT.

#### 2.3.2. GSEA–Gene Ontology (GO) Annotation Analysis of LUT Treating LOD

**Figure 5:**
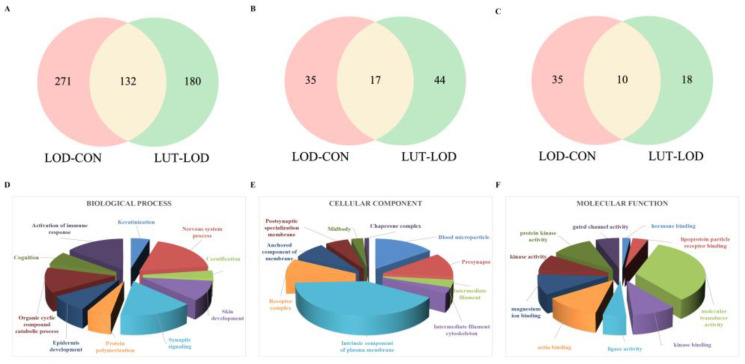
Gene Ontology (GO) annotation results for LUT treatment of LOD derived from CSF proteomics data. (**A**) GO annotation results for biological processes (BPs); (**B**) GO annotation results for cellular components (CCs); and (**C**) GO annotation results for molecular functions (MFs). (**D**) A total of 132 GO-BPs were co-regulated by LOD/CON and LUT/LOD. The *p*-values of GO-BPs for the above LUT/LOD were ranked, and the top ten significant GO-BPs were then displayed in pie diagrams. (**E**) A total of 17 GO-CCs were co-regulated by LOD/CON and LUT/LOD. The *p*-values of GO-CCs for the above LUT/LOD were ranked, and the top ten significant GO-CCs were then displayed in pie diagrams. (**F**) A total of 10 GO-MFs were co-regulated by LOD/CON and LUT/LOD. The *p*-values of GO-MFs for the above LUT/LOD were ranked and displayed in pie diagrams.

In CSF proteomics data, GSEA was used for the GO annotation of genes jointly enriched in the LOD and CON groups. This produced 500 results, of which 403 items were biological processes (BPs), 52 items were cellular components (CCs) and 45 items were molecular functions (MFs). The GSEA–GO method was adopted to annotate the genes co-enriched in the LUT and LOD groups, and 252 items were obtained. Among these items, the BP, CC, and MF categories contained 192, 43, and 17 items, respectively (*p* < 0.1) (Figure 5A–C).

Matching the GO annotation outcomes of LOD/CON and LUT/LOD, we found that they commonly modulated BPs (132 items), CCs (17 items), and MFs (10 items). It was concluded that LUT might exert a therapeutic effect on LOD mainly through the regulation of synaptic plasticity, neurogenesis, cognition, axonogenesis, aging, and other biological processes of genes. Furthermore, LUT primarily interfered with cellular components such as the postsynaptic density membrane, spindle, and presynapse, and molecular functions such as cytoskeletal protein binding and calcium-dependent protein binding, thus functioning as a therapy for LOD (Figure 5D–F).

#### 2.3.3. Analysis of the Core GSEA–KEGG Pathways for LOD Treatment by LUT

**Figure 6:**
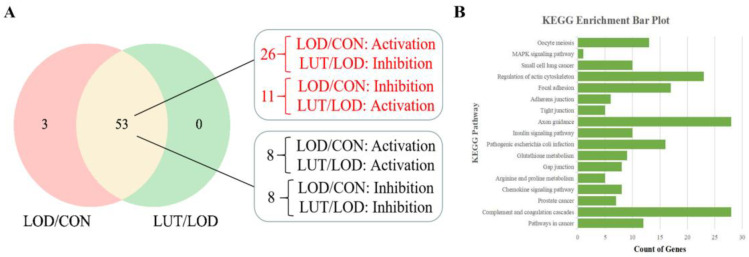
Analysis of the GSEA–KEGG/KEGG pathways for LOD treatment by LUT. (**A**) GSEA–KEGG pathway enrichment in LUT treatment on LOD generated from CSF proteomics data. (**B**) The GSEA–KEGG results of CSF proteomics were identical to 17 pathways in the KEGG results of network pharmacology. The 17 pathways are named as shown in the figure.

An analysis of the CSF proteomics data through GSEA revealed that the proteins co-identified by the LOD and CON groups were enriched to 56 GSEA–KEGG pathways. Similarly, the proteins identified in the LUT group together with the LOD group were enriched to 53 GSEA–KEGG pathways. The GSEA–KEGG results for both were matched, and a common enrichment was found in 53 pathways. In total, 26 GSEA–KEGG pathways were activated in the LOD group (vs. the CON group) while being inhibited in the LUT group (vs. the LOD group). In addition, there were 11 GSEA–KEGG pathways in an inhibited state in the LOD group (vs. the CON group), concurrent with the LUT group in an activated state (vs. the LOD group). The 37 GSEA–KEGG pathways mentioned above were mainly involved in glutathione metabolism, tight junctions, actin cytoskeleton regulation, and axon guidance (Figure 6A).

The network pharmacology results displayed 1282 targets for “Aging”, 1178 targets for “Depression”, and 436 targets for LUT. The targets of “Aging” and “Depression” were combined to generate 2181 target genes of LOD. The target genes of LUT and LOD were cross-matched, and they were found to co-regulate 166 genes. These 166 genes were input into the DAVID database for KEGG pathway analysis, which ultimately yielded 179 KEGG pathways.

The 37 GSEA–KEGG pathways derived from proteomics were paired with the 179 KEGG pathways acquired from network pharmacology. The results demonstrated that 17 GSEA–KEGG/KEGG pathways were identical, including axon guidance, glutathione metabolism, and actin cytoskeleton regulation (Figure 6B).

In the CSF proteomics data, the core genes of LOD/CON in the above 17 GSEA-KEGG/KEGG pathways can be obtained by GSEA, and likewise for LUT/LOD. Individually, these core genes were compared to 133 differentially expressed genes detected by CSF proteomics. It was revealed that only the proteins corresponding to the core genes in the axon guidance pathway were affiliated with 133 differentially expressed proteins. The proteins corresponding to the core genes in the remaining 16 GSEA–KEGG/KEGG pathways could not be matched to these 133 differentially expressed proteins. 

#### 2.3.4. HUB Gene Analysis of the Treatment of LOD by LUT through a Protein–Protein Interaction (PPI) Network

**Figure 7:**
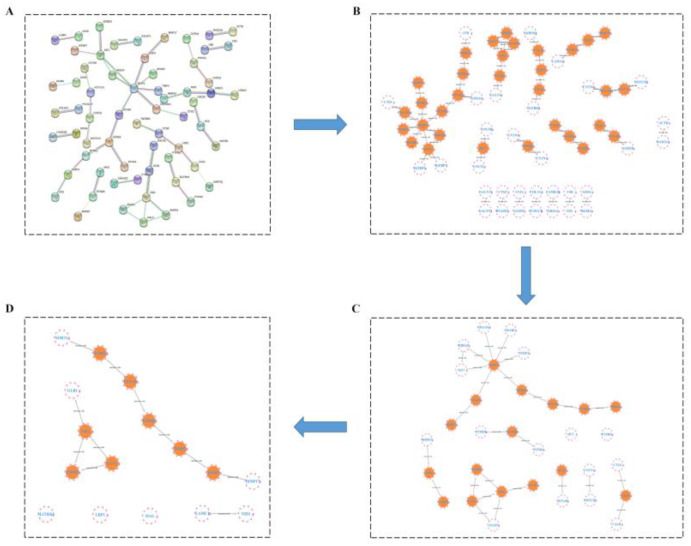
Protein–protein interaction (PPI) network analysis of differentially expressed proteins between luteolin and LOD from CSF proteomics data. (**A**) PPI diagram showing the interaction of differentially expressed proteins from LUT versus LOD, with a minimum confidence level set at 0.9. (**B**–**D**) Topology analysis using the CytoNCA plug-in of Cytoscape software identified a total of eight HUB genes after three filters for the 64 nodes of the PPI network.

After importing 243 differentially expressed genes from the LOD and LUT groups into the String website, we obtained a target–target interaction network involving 64 nodes and 50 edges (Figure 7A). Genes greater than the median of all nodes were screened according to their betweenness (BC), closeness (CC), and degree (DC). Altogether, eight HUB genes of LUT for LOD were obtained after three filters: namely, *SMPD1*, *EPHB4*, *GBA*, *MMP2*, *HBEGF*, *EFNA5*, *GALC*, and *IGF-1* (Figure 7B–D).

Surprisingly, we discovered that the proteins corresponding to *EPHB4* and *EFNA5* were also differentially expressed proteins in the axon guidance pathway. This sufficiently suggested that the axon guidance pathway might be the core pathway for the LUT treatment of LOD. Hence, we will highlight the potential targets of LUT for LOD treatment from the axon guidance pathway.

### 2.4. Analysis of Potential Targets for LUT Treatment on LOD Based on Axon Guidance Pathway

#### 2.4.1. GSEA–KEGG Results of Axon Guidance Pathway Based on CSF Proteomics

**Figure 8:**
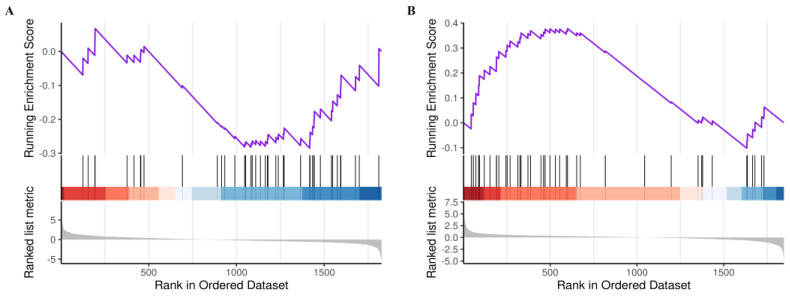
GSEA–KEGG analysis of axon guidance pathway. (**A**) LOD versus CON. (**B**) LUT versus LOD.

In comparison with the CON group, the axon guidance pathway was in a suppressed state in the LOD group. It involved a series of 28 genes such as *PLXNB2*, *EFNA1*, *EPHB2*, and *SEMA3C*. (Figure 8A). Upon LUT treatment, the axon guidance pathway was in an activated state. This involved a sum of 26 genes such as *EPHA7*, *SEMA4B*, *EFNA5* and *UNC5B* (Figure 8B).

#### 2.4.2. Analysis of Potential Targets of LUT for LOD Based on Axon Guidance Pathway

**Figure 9:**
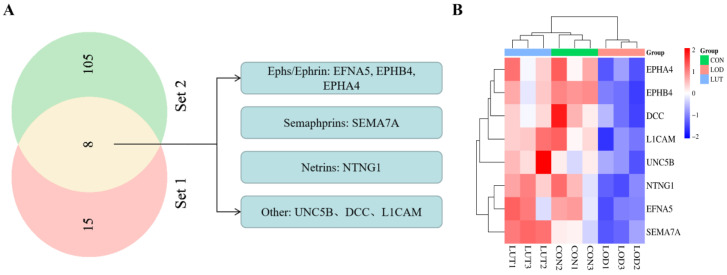
Eight potential targets on the axon guidance pathway for the LUT treatment of LOD. (**A**) Eight potential targets of LUT for LOD. Five of these targets belonged to the four major families of axon guidance molecules, namely the Ephs/Ephrin family, Semaphorins family, and Netrins family. Set 1: Twenty-three genes involved in LOD/CON and LUT/LOD in the axon guidance pathway are identical. Set 2: CSF proteomics yielded 113 differentially expressed proteins for LUT treatment of LOD. (**B**) Heat map demonstrating protein expression levels of eight potential targets for LUT treatment of LOD, with red indicating upregulation and blue indicating downregulation.

The genes involved in LOD/CON and LUT/LOD in the axon guidance pathway were compared, and 23 genes were found to be identical. These included *EFNA5*, *EPHA4*, *EPHB4*, *UNC5B*, *NTNG1*, and *DCC*. Out of these genes, 8 genes could be matched to 113 differentially expressed genes obtained by CSF proteomics, specifically *EFNA5*, *EPHA4*, *EPHB4*, *UNC5B*, *NTNG1*, *DCC*, *SEMA7A*, and *L1CAM* (Table 1). We hypothesized that these eight genes may be potential targets on the axon guidance pathway for the LUT treatment of LOD. Regarding these eight genes, five genes belonged to the four major axon guidance molecules families, i.e., the Ephs/Ephrin family (*EFNA5*, *EPHB4*, and *EPHA4*), the Semaphorins family (*SEMA7A*), and the Netrins family (*NTNG1*) (Figure 9A,B).

#### 2.4.3. Molecular Docking Results of LUT with Eight Potential Targets on the Axon Guidance Pathway

**Figure 10:**
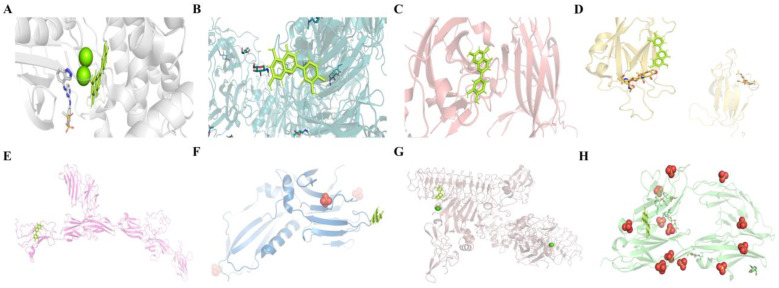
Molecular docking results of LUT with eight potential targets on the axon guidance pathway: (**A**) LUT and *EPHB4*; (**B**) LUT and *SEMA7A*; (**C**) LUT and *EPHA4*; (**D**) LUT and *EFNA5*; (**E**) LUT and *L1CAM*; (**F**) LUT and *UNC5B*; (**G**) LUT and *NTNG1*; and (**H**) LUT and *DCC*.

Molecular docking techniques between small molecules and targets can predict the binding mechanism and activity between active ingredients and target proteins to some extent [19]. It was reported that docking values less than −4.25 k/mol indicated a moderate binding activity, less than −5.0 k/mol indicated a good binding activity, and less than −7.0 k/mol indicated a strong binding activity [20]. The results demonstrated that LUT had a strong binding activity with *EPHB4*, *SEMA7A*, and *EPHA4*, and a better binding activity with *EFNA5*, *L1CAM*, *UNC5B*, *NTNG1*, and *DCC* (Table 2). The lower ΔG indicated a more stable binding activity between the small molecules and the targets, thereby suggesting a high affinity of LUT for these eight potential targets. Finally, we applied the Pymol software to visualize the docking results of LUT with the above potential targets (Figure 10).

## 3. Discussion

Depression is a prevalent mental health problem that affects older individuals. Clinical studies have revealed that LOD patients suffer from impairments in information processing and executive function [21]. Cognitive deficits are distinct characteristics of LOD, which are also high risk factors of developing Alzheimer’s disease [22]. Currently, the treatments for LOD include electroconvulsive therapy, repetitive transcranial magnetic stimulation, vagus stimulation, and psychotherapy, etc. [23]. Nevertheless, there are adverse responses to treatment, such as treatments being poorly tolerated by patients or strong side effects. Consequently, finding effective treatments to reduce the morbidity and mortality of LOD remains an imperative issue.

Over thousands of years, we have received a large number of biologically active molecules with medicinal properties from nature, and the identification of their molecular targets and action mechanisms is the current focus of natural product research [17]. LUT is among the most prevalent naturally polyphenolic flavonoid compounds. It is available in honeysuckle, chrysanthemum, perilla, scutellaria, and other traditional Chinese medicines [24]. The current biologics of LUT are mainly consumed orally, but have a low absorption rate of about 15% [11]. Adverse reactions were observed when LUT was used as a dietary supplement; however, the incidence of such reactions was relatively low [25]. In consideration of the above characteristics, a variety of LUT formulations are being developed. For instance, liposomal preparations using olive pomace oil [26], dietary formulations with LUT [27], and ultra-micronized preparations [28] can all enhance the oral absorption and safety of LUT. Previous studies have shown that LUT possesses biological activities such as neuroprotection, anti-neuroinflammation, and the prevention of neuronal death [16,29]. LUT holds great promise for the treatment of brain-related disorders such as cerebral ischemia, Alzheimer’s disease, depression, and autism spectrum disorders [30,31,32]. Future experimental studies and clinical trials on LUT can further expand the pharmacological mechanisms and clinical applications of LUT. 

Proteins constitute the majority of biochemically active components in biological systems and are the targets of almost all drugs [33]. Proteomics-based approaches provide unbiased, high-throughput, and quantitative results that can be useful for studying proteins of concern [17]. In comparison to conventional KEGG, GSEA does not necessitate the designation of explicit differential gene thresholds. GSEA is more likely to encompass subtle but synergistically varied biological pathways from the perspective of gene set enrichment, especially those with small differential multiplicities [34]. Natural compounds are not chemically modified, and have mild and persistent actions as well as a wide range of targets. Thus, it is quite appropriate to adopt GSEA for investigating the possible pathways and potential targets of LUT for LOD treatment. Molecular docking simulations predict affinity and binding modes through ligand–receptor interactions, which helps to provide a reliable basis for experiment-based assays, thereby accelerating drug design and selection [35]. Consequently, mechanistic studies of LUT against LOD, based on proteomics, GSEA, and molecular docking to simulate natural compound–target binding, can provide a foundation for the development of targeted drugs for LOD.

In our study, the core GSEA–KEGG pathway for the LUT treatment of LOD was the axon guidance pathway. We reviewed the literature with “depression” and “axon guidance” as subject terms. It was found that most genes in the axon guidance pathway were enriched in either depression or CNS diseases. In a genome-wide association study of depression-related genes, axon guidance molecular genes (netrins, slits, semaphorins, ephrins, and cell adhesion molecules) were found to be significantly enriched in depressed patients [36]. In a hippocampal transcriptomics study of patients with major depressive disorder vs. an animal model, a functional analysis of differentially expressed genes revealed that the axon guidance signaling pathway was the most prominently enriched [37]. The previous perception considered aging to be a confounding factor in chronic disease, which could then be ignored. However, as the underlying biological mechanisms of aging are investigated, the discovery of interventions to prolong aging and prevent the onset of chronic diseases is promising [38]. From a study on plasma proteomics in healthy populations, 217 proteins exhibited a high correlation with age. It was also observed that these proteins were notably enriched in the axon guidance pathway [39]. Taken together, the axon guidance pathway and the genes in this pathway play an essential part in depression and aging.

During the development of the nervous system in animals, the axonal growth and extension of neurons follow a rigorous direction [40]. Axon guidance is a necessary process in the formation of neural circuits. Axons locate the growth direction in the developing nervous system to reach the appropriate neuron targets. The clues that guide axons to form synapses are known as axon guidance molecules [41]. Axon guidance molecules participate in processes, such as somatic axis formation and axon growth, which play an essential role in the growth and maturation of the nervous system [42]. Axon guidance molecules consist of four major families of protein molecules: namely, the Slits, Semaphorins, Netrins, and Ephs families. The core GSEA–axon guidance pathway and eight potential targets (three from the Ephs family and one each from the Semaphorins and Netrins families) for the LUT treatment of LOD were identified in this study using proteomics and molecular docking approaches (see below). Although we emphasize the role of various guidance molecules in modulating growth and guiding axons, extensive studies have shown that they also regulate neuronal migration, synapse formation, axon pruning, neuronal death, and axon regeneration in the nervous system [43]. As such, we believed that axon guidance pathways might exert a broader effect in the treatment of LOD by LUT.

Ephs family: ephrin/Eph signaling governs dendritic spine morphogenesis in neurons and synaptogenesis in adult hippocampal neurons [44]. The dysfunction of ephrin-A5 leads to a reduced proliferation of neural stem cells (NSCs) and the impaired survival/maturation of newborn neurons in the dentate gyrus of the adult hippocampus [45]. Ephrin-B2/EphB4 signaling promotes neuronal differentiation by activating β-catenin and inducing the transcription of neurogenic factors [46]. *EphB4* knockdown suppresses the differentiation of embryonic NSCs to neurons, while *EphB4* overexpression facilitates the auto-renewal and proliferation of NSCs [47]. EphA4 possesses the capacity to broadly conjugate the ligands from the ephrin A and B family, as well as to maintain NSC proliferation [48]. EphA4 performs essential functions in the developing and postnatal brain, including the modulation of axonal guidance, synaptic plasticity, and neurogenesis [49,50].

Semaphorins family: Members of the semaphorin family are widely distributed in the nervous system and are bound to influence processes such as axon guidance, axon branching, and synapse formation [51]. Sema7A is a membrane-anchored member of the Semaphorin family that enhances central and peripheral axon growth while being required for the formation of axon bundles during embryonic progression [52]. 

Netrins family: Netrin-G1 (NTNG1) is a specific axon guidance molecule in vertebrates, which is primarily located on developing axons. It was shown that NTNG1 KO mice were deficient in the learning phase that regulated spatial reference information and working memory [53]. NTNG1 is crucial in neural development, including the promotion synapse formation, axon growth and the regulation of synaptic plasticity. Moreover, NTNG1 deficiency contributed to fear-like and anxiety-like behavioral disturbances [54].

Unc5B is a ligand-dependent receptor that fosters cell survival in the presence of Netrin-1 while inducing cell death in the absence of a ligand [55]. Neural cell adhesion molecule L1 (L1CAM) plays an essential role in neural development, regulating processes such as neurite growth, cell migration, neuronal survival, and synaptic plasticity [56]. In humans, L1CAM mutations may cause severe neurodevelopmental defects [57]. DCC is expressed on axons and growth spines, and is also the cell surface receptor for Netrin-1. The Netrin-1/DCC interaction is not only essential for neuronal development but also plays a part in multiple cellular processes, including cell adhesion, proliferation, differentiation, and cell survival [58]. The binding of Netrin-1 to DCC mediates axon guidance and is coupled with directing axon growth and cell migration [59].

LUT may exert its therapeutic effects on LOD by modifying CSF components. The CSF constituents here mainly refer to the genes of the three major axon guidance molecule families and their corresponding receptors. The literature research revealed that no studies have reported the exertion of therapeutic effects of LUT by modulating axon guidance molecules. The prevailing investigations also failed to explore the mechanism of LOD from this viewpoint. Our previous studies have shown that LUT can improve depressive-like behavior and cognitive impairment in LOD rats by enhancing folate brain transport and increasing folate content in the CSF [60]. Apart from this, no other reports were found to explore the pharmacological mechanisms of LUT from the perspective of modified CSF constituents. Therefore, this study presents new insights and directions for the pharmacological study of LUT and the discovery of new drugs for LOD.

By integrating proteomics (GSEA), a PPI network analysis, and molecular docking, we discovered the core axon guidance pathway of LUT for LOD treatment and identified eight potential targets on this pathway. Nevertheless, an awareness of the limitations of the proteomics approach should be maintained. Additionally, we did not conduct a subsequent experimental validation of the potential targets, making the study results somewhat unconvincing. Moreover, adult normal rats (the CON group) were selected as the control group (instead of naturally aging rats) to LOD rats. This was mainly because the effects of LUT should not be limited to anti-depression but should also include some anti-cerebral-aging sessions. The emphasis of this study was not to explore the pathological mechanisms of LOD; therefore, no subgroups of the analytic design were performed. This is somewhat defective, and we will compensate for it in future studies. Still, this establishes a foundation for further studies of LUT, which will be one of our future research directions.

## 4. Materials and Methods

### 4.1. Animals

The experimental protocol was approved by the Animal Ethics Committee of the Guangzhou University of Traditional Chinese Medicine. All experiments were performed according to the guidelines for the care and use of laboratory animals of the Animal Experimentation Center of the Guangzhou University of Chinese Medicine. 

Male Wistar rats at 8 months of age were purchased from Beijing Vital River Laboratory Animal Technology Co., Ltd., Beijing, China (license No.: SCXK 2016-0011) and raised in the SPF barrier system until 20 months of age. Male Wistar rats at 7–8 weeks of age were purchased from Southern Medical University Laboratory Animal Center (license No.: SCXK 2016-0041).

The rats were removed from the experiment by the sucrose preference test (SPT). The criteria for removal were a low sucrose preference (an SP less than 60%), location preference (preference for liquids with fixed orientation), low water consumption (neither sucrose nor pure water), and excessive water consumption (total fluid consumption of more than twice the mean value of total fluid consumption of all rats). All rats were reared in a separate cage during the SPT.

All eligibly aged rats were randomly divided into the LOD group and the LUT group based on body weight and SP, with 12 rats in each group. Similarly, 12 young, normal rats were marked as the CON group. All rats were fed ad libitum on a light/dark cycle of 12 h/12 h (light on 08:00–20:00) and at a temperature of 23 ± 2 °C. The experimental protocol was approved by the Animal Ethics Committee of the Guangzhou University of Traditional Chinese Medicine. All experiments were performed according to the guidelines for the care and use of laboratory animals of the Animal Experimentation Center of Guangzhou University of Chinese Medicine. 

### 4.2. Chronic Unpredictable Mild Stress (CUMS) Modeling

Rats in the CON group were kept in room A without any stimulation, with four rats per cage. Rats in both the LOD and LUT groups were subjected to chronic, unpredictable mild stress. They were maintained in Room B and fed separately. The stressors consisted of fasting (12 h), water deprivation (12 h), constant illumination (24 h), foot shock (1 mA, 2 s/time, 10 times), white noise (85 dB, 5 h), stroboscopic illumination (300 times/min, 5 h), thermal swimming (45 °C, 5 min), restraint (12 h), humidified cage (10 h), fasting and abstaining (24 h), and being housed with another four rats (10 h). Rats were randomly exposed to 1–2 stressors once a day for six weeks with no stimuli, and this was repeated for more than three consecutive days.

**Figure 11:**
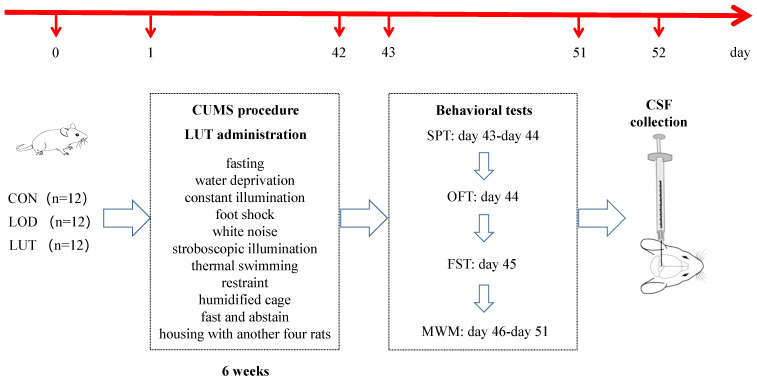
Experimental timeline. LOD: late-onset depression; LUT: luteolin; CUMS: chronic unpredictable mild stress; SPT: sucrose preference test; OFT: open field test; FST: fast swimming test; MWM: Morris water maze test; and CSF: cerebrospinal fluid.

Rats in the CON and LOD groups received saline gavage once daily at a dose of 4 mL/kg. Rats in the LUT group were given luteolin solution by gavage at a dose of 25 mg/kg once daily. The dose selection of LUT was based on previous studies that reported the neuroprotective, anti-inflammatory, and anti-apoptotic effects of LUT [61,62]. The LUT was obtained from Nanjing Dilger Medical Technology Co., Ltd. and identified by high-performance liquid chromatography with a purity ≥ 98% (Figure 11). 

### 4.3. Behavioral Tests

#### 4.3.1. Sucrose Preference Test (SPT)

The SPT was used to detect anhedonia in rats. The SPT was classified into four phases: sucrose training for 48 h, a baseline test for 36 h, fasting and water deprivation for 24 h, and a sucrose preference test for 12 h. In the final stage, two bottles of liquid (1% sucrose solution and pure water) were given simultaneously to each animal, and the bottles were removed after 12 h of liberal drinking. The bottles were weighed, and the sucrose preference was calculated. [Sucrose preference (%) = sucrose consumption/total liquid consumption × 100). Following 6 weeks of modeling, all rats were deprived of food and water for 24 h. The SPT was conducted for 12 h and the post-modeling sucrose preference was then calculated.

#### 4.3.2. Open Field Test (OFT)

The open field test was used to evaluate autonomous mobility and anxiety in rats. Prior to the experiment, all rats were transferred into a behavioral test room (a soundproof, dark room) for 1 h to become acclimatized to the environment. For the formal experiment, the rats were gently placed into the center of the box (100 cm × 100 cm × 48 cm). Following approximately 10 s of adaptation, the activities of the rats in the wooden box were recorded for 5 min with a camera analysis system (Guangzhou FIDI Company). Among the activities, the number of grid crossings and the overall distance were used to assess autonomic movements. The distance and time spent in the central zone were applied to evaluate anxiety.

#### 4.3.3. Forced Swimming Test (FST)

The forced swimming test was used to appraise the anhedonia of the rats. A cylindrical, transparent water bucket was used, which measured 30 cm in inner diameter and 100 cm in height and contained water at a depth of 35 cm and a temperature of 25 ± 1 °C. The rats were familiarized with the environment by moving them into the test room 1 h prior to the experiment. During the trial, the rats were slowly put into the swimming bucket and the video equipment was employed to capture the rats’ movements within 6 min. A double-blind recording of the time of immobility behavior of the rats in the barrel from the third to the sixth min was employed. A double-blind procedure was carried out to record the immobility time of the rats from the 3rd to the 6th min. The criterion for immobility was a rat floating on the water, with inactive limbs or a slight paddling of the front paws and tail to keep the head above the water.

#### 4.3.4. Morris Water Maze Test (MWM)

The Morris water maze test was used to estimate the learning and spatial memory function of the rats. The pool was split equally into four quadrants, with the platform located in the center of any quadrant. Video equipment was installed above the maze to track the movement of the rats. The water temperature was regulated at 25 ± 2 °C. The entire experiment comprised two steps and lasted for a combined 6 days. The orientation navigation test lasted for 5 d with four training sessions per day, each 30 min apart. For training, the investigators randomly selected one quadrant as the water entry point and plunged the rats into the water, allowing them to explore liberally for 2 min. Once the rats had reached the platform, their escape latency was documented, and they were allowed to settle on the platform for 10 s. If the plateau was not identified within 120 s, the latency was marked as 120 s, and the rats were guided to stay on the platform for 10 s. For the spatial exploration test, the platform was withdrawn on the sixth day. The rats were plunged into the water facing the wall of the pool at any entrance point, and their motion was observed within 120 s. The detection indexes were the escape latency, the number of platform crossings, and the time ratio of the quadrant in which the platform was located.

### 4.4. CSF Sample Collection

Immediately after the rats were anesthetized (10% chloral hydrate, 3.5 mL/kg, intraperitoneally), their heads were fixed, and the skin and muscles were clipped to expose the foramen magnum. The CSF was collected by puncture extraction from the cerebellomedullary cistern using a 1 mL syringe attached to a 0.45 gauge needle [61]. The acquired cerebrospinal fluid was placed on ice for 20 min, then centrifuged at 4 °C for 15 min at 3000 rpm, and the supernatant was gathered and preserved at −80 °C.

### 4.5. CSF Proteomics Analysis

#### 4.5.1. Analysis of CSF Proteomics with Data Independent Acquisition (DIA) Method

The total protein was isolated from the CSF samples, and protein quantification was performed using the BCA method. From each individual sample, 20 µg of protein was added to a 6 × loading buffer, proceeded to SDS-PAGE electrophoresis, and stained with Kaomas Brilliant Blue. Peptides were fractionated and quantified from 200 µg of the protein solution using the FASP enzymatic digestion protocol. All peptide mixtures were classified by the Agilent 1260 infinity II HPLC system. An amount of 1 µg of peptide was taken from each fraction, mixed with iRT peptide, then separated by nano-LC. The raw mass spectrometry data were analyzed by a Spectronaut Pulsar X (Biognosys AG), and a spectral database was developed.

To ensure the accuracy of quantification, the DIA results were processed with a normalization strategy. Data with at least half of the nonsense values within either group were selected for significant difference analysis. DEPs were proteins whose fold change was more than 1.2-fold and who had a p value of less than 0.05.

#### 4.5.2. GSEA for GO Annotation and KEGG Pathway Enrichment

1. The proteins were identified commonly by the CON and LOD groups, and the LUT and LOD groups were GO-annotated employing the GSEA method;
2. The GSEA method was applied for KEGG pathway enrichment from the co-identified proteins of the CON and LOD groups and the LUT and LOD groups;
3. The activity of the GSEA–KEGG pathways was determined based on the enrichment score, with less than zero indicating inhibition and greater than zero indicating activation. We screened the GSEA–KEGG pathway, which is co-regulated by LOD/CON and LUT/LOD with opposite activity states, as the key pathway for the LUT treatment of LOD.

#### 4.5.3. Protein–Protein Interaction (PPI) Network Analysis

DEPs of the CSF proteomics data from the LUT and LOD groups were converted for gene identification in the DAVID database. The above differential genes were analyzed using the String website. With the species set to “homo sapiens”, the minimum confidence level set to 0.9, and the free nodes hidden, we obtained the results of the PPI network analysis. The PPI file was imported into Cytoscape 3.9.1 software and a topology analysis was performed using the CytoNCA plug-in. Genes greater than the median of all nodes were sorted according to their betweenness (BC), closeness (CC), and degree (DC).

#### 4.5.4. Molecular Docking Experiments

The InChI Key of LUT on the PubChem website was searched, and the mol2 file of LUT from the TCMSP website was downloaded. The PDB file of the target protein from the RCSB PDB website was downloaded. If no appropriate PDB structure was available, the Swiss model website was adopted for homologous modeling to screen the best template with GMQE (global model quality estimate).

The PDB file was manipulated in the Pymol software to remove the ligands and non-protein molecules (e.g., water molecules). The structure was then repaired using the https://swift.cmbi.umcn.nl/servers/html/model.html (accessed on 12 July 2022) website. The mol2 file of LUT and the PDB file of the target protein were, respectively, introduced into the Swissdock software for molecular docking to obtain the binding energy ΔG (kcal/mol). Pymol software was eventually utilized to visualize the molecular docking results.

### 4.6. Network Pharmacology Analysis

#### 4.6.1. Target Prediction of LUT 

TCMSP, Swiss Target Prediction, SymMap, PharmMapper, ChEMBL, HERB, and ETCM databases were used to recruit targets for LUT. Among them, the targets captured from the TCMSP website were genetically normalized by the Uniprot database to delete unannotated and duplicate targets. The confidence level of ChEMBL was set to 80% to scan the targets of active status. In all the above-mentioned databases, species were defined as “human”.

#### 4.6.2. Target Genes for LOD

1. We searched for depression-related targets using the GeneCards, DisGenet, OMIM, TTD, PharmGKB, and DrugBank databases, with the terms “depression” or “major depressive disorder” (hereafter collectively referred to as “Depression”) used as keywords. The targets generated from the GeneCards database were filtered with a relevance score ≥ 5. The targets acquired from the DisGeNET database were screened with a score ≥ 0.3.
2. Aging-related targets were investigated in the GeneCards, DisGenet, OMIM, TTD, PharmGKB, and DrugBank databases based on the keywords “aging”, “senescence”, or “anti-aging” (hereafter collectively referred to as “Aging”). Targets derived from the GeneCards database were sorted by a relevance score ≥ 5. Targets obtained from the DisGeNET database were screened by a score ≥ 0.3.
3. The target genes of “Depression” and “Aging” were merged to produce the target genes of LOD.

#### 4.6.3. Network Pharmacology Analysis of the KEGG Pathway in LUT Treatment of LOD

The intersection genes of LUT and LOD were screened, and these genes were imported into the DAVID database for KEGG pathway enrichment. The significance of gene enrichment in the KEGG pathway was compared via Fisher’s exact test, and the results were ranked by significance level.

### 4.7. Statistical Analysis

SPSS 22.0 software was applied for the statistical analysis of all the experimental data. A one-way analysis of variance (ANOVA) was performed for each group of data if they followed a normal distribution (Shapiro–Wilk method test). In pairwise comparisons, the LSD method was employed for those meeting the homogeneity of variance, and the Games–Howell method was conducted for those with non-homogeneous variance. If some data sets did not conform to a normal distribution, a nonparametric test was carried out. A two-way repeated measurement ANOVA was used to analyze the escape latency of the Morris water maze, and those that did not satisfy Mauchly’s spherical test were corrected with the Greenhouse–Geisser method. All data were expressed as the mean ± SEM, with *p* < 0.05 being considered statistically significant between groups.

## 5. Conclusions

LUT may exert its therapeutic effects on LOD by modifying CSF components. The CSF constituents here mainly refer to genes of the three major axon guidance molecule families and their corresponding receptors. Specifically, *EFNA5*, *EPHB4*, *EPHA4*, *SEMA7A*, *NTNG1*, as well as *UNC5B*, *DCC*, and *L1CAM* may be potential targets for the LUT treatment of LOD. 

## Figures and Tables

**Table 1:** Eight potential targets on the axon guidance pathway for LUT treatment of LOD.

Protein Name	Gene Name	Family	LOD/CON		LUT/LOD	
			Fold Change	*p* Value	Fold Change	*p* Value
Ephrin-A5	*EFNA5*	Ephs/Ephrin	0.57456	0.01075	1.85970	0.02264
EPH receptor B4	*EPHB4*		0.56399	0.00026	1.54778	0.00798
Eph receptor A4	*EPHA4*		0.69904	0.01011	1.38487	0.01475
Semaphorin 7A	*SEMA7A*	Semaphorins	0.81877	0.01265	1.46891	0.00033
Netrin G1	*NTNG1*	Netrins	0.74603	0.01773	1.37943	0.00113
Netrin receptor UNC5B	*UNC5B*	Other	0.80701	0.02041	1.47754	0.03082
Neural cell adhesion molecule L1	*L1CAM*		0.78713	0.01719	1.28610	0.00572
Netrin receptor DCC	*DCC*		0.66085	0.02909	1.33989	0.01489

LUT: luteolin; LOD: late-onset depression.

**Table 2:** Molecular docking scores of luteolin with eight potential targets on the axon guidance pathway.

Compound	Target Name	Protein Name	ΔG (kJ·mol^−1^)	Target Name	Protein Name	ΔG (kJ·mol^−1^)
Luteolin	*EPHB4*	EPH receptor B4	−8.0970	*L1CAM*	Neural cell adhesion molecule L1	−6.9822
	*SEMA7A*	Semaphorin 7A	−7.3077	*UNC5B*	Netrin receptor UNC5B	−6.9678
	*EPHA4*	Eph receptor A4	−7.0616	*NTNG1*	Netrin G1	−6.8523
	*EFNA5*	Ephrin-A5	−6.9858	*DCC*	Netrin receptor DCC	−6.6671

## Data Availability

The mass spectrometry proteomics data have been deposited into the ProteomeXchange Consortium via the PRIDE partner repository with the dataset identifier PXD038159.

## Abbreviations

CSF: cerebrospinal fluid; DCC: netrin receptor DCC; DEPs: differentially expressed proteins; EFNA1: Ephrin-A1; EFNA5: Ephrin-A5; EPHA4: Eph receptor A4; EPHA7: Ephrin type-A receptor 7; EPHB2: Eph receptor B2; EPHB4: EPH receptor B4; GALC: galactosylceramidase; GBA: Glucosylceramidase; HBEGF: heparin-binding, EGF-like growth factor; IGF-1: insulin-like growth factor 1; L1CAM: neural cell adhesion molecule L1; LOD: late-onset depression; LUT: luteolin; MMP2: matrix metallopeptidase 2; NTNG: netrin G1; PLXNB2: Plexin B2; SEMA3C: Semaphorin 3C; SEMA4B: Semaphorin 4B; SEMA7A: Semaphorin 7A; SMPD1: sphingomyelin phosphodiesterase 1; and UNC5B: netrin receptor UNC5B.
